# Osimertinib-induced severe bilateral pneumothorax: A case report

**DOI:** 10.1097/MD.0000000000036994

**Published:** 2024-01-19

**Authors:** He Li, Xiaojuan Shi, Gang Chen, Dongchang Wang

**Affiliations:** a Department of Respiration, The Third Hospital of Hebei Medical University, Shijiazhuang, China.

**Keywords:** adverse event, non-small cell lung cancer (NSCLC), osimertinib, pneumothorax, tyrosine kinase inhibitor (TKI)

## Abstract

**Rationale:**

Osimertinib is the third-generation, pyrimidine-based, irreversible epidermal growth factor receptor-tyrosine kinase inhibitor that received approval from the FDA in November 2015 and has become the standard approach in patients with advanced, epidermal growth factor receptor (EGFR) mutated non-small cell lung cancer (NSCLC), especially with brain metastases. Osimertinib is beneficial in terms of progression-free and overall survival in patients with EGFR-mutated NSCLC. However, the rarity of bilateral pneumothorax among adverse events necessitates further research on its potential fatality rate.

**Patient concerns:**

A 72-year-old man diagnosed with stage IV (T2NxM1) NSCLC with the 21L858R mutation of the EGFR gene received osimertinib treatment. Unfortunately, 10 weeks after osimertinib treatment, the patient developed severe interstitial lung disease and pneumothorax. Thus, osimertinib treatment was discontinued, and prednisolone (160 mg/day) and supportive treatment were administered.

**Diagnoses:**

Osimertinib-induced severe interstitial lung disease and pneumothorax.

**Interventions:**

Osimertinib treatment was discontinued, and prednisolone (160 mg/day) and supportive treatment were administered.

**Outcomes:**

The bilateral pneumothorax was difficult to correct and the patient eventually died.

**Lessons:**

Osimertinib-induced pneumothorax occurred approximately 10 weeks after receiving the drug and had severe cough and chest tightness as initial symptoms. In addition, the incidence of drug-induced pneumothorax increases in patients treated with osimertinib when combined with underlying respiratory diseases.

## 1. Introduction

Lung cancer is the most prevalent malignant tumor and the primary cause of cancer-related deaths worldwide, with non-small cell lung cancer (NSCLC) accounting for 80% of all lung cancer cases.^[1–3]^ NSCLC is the most common histological type and has a poor prognosis. Stage IV NSCLC is associated with a 5-year survival rate of ~1%.^[4]^ The discovery of driver mutations has drastically changed clinical practice in patients with lung tumors. Multiplexed testing contributes to physicians’ selection of therapies; individuals with drivers receiving a matched targeted agent live longer at the same time.^[5]^ Osimertinib is a third-generation irreversible oral epidermal growth factor receptor-tyrosine kinase inhibitor (EGFR-TKI) that selectively inhibits both EGFR-TKI-sensitizing and epidermal growth factor receptor (EGFR) T790M resistance mutations.^[6,7]^ The previous trials have shown that osimertinib is associated with significantly improved efficacy and a favorable safety profile in patients with EGFR mutation-positive, locally advanced, or metastatic NSCLC, both as a first-line treatment and as a second-line treatment for progression after first-line EGFR-TKI treatment.^[8,9]^ Moreover, EGFR T790M mutation-positive advanced NSCLC patients with poor performance status could benefit from osimertinib therapy.^[10]^ However, in addition to providing an excellent survival benefit, tyrosine kinase inhibitors (TKIs) can also induce adverse events, which is a major management challenge for patients with advanced NSCLC. TKI-related adverse events such as diarrhea, paronychia, rash, decreased platelet count, decreased appetite, interstitial lung disease (ILD), and pneumothorax are commonly reported.^[11–13]^ Drug-related lung damage, including drug-related pneumonia, pneumothorax, and diffuse alveolar hemorrhage, is a particularly worrisome adverse event. Herein, we report a case of lung adenocarcinoma with multiple metastases that developed severe bilateral spontaneous pneumothorax when treated with osimertinib as first-line treatment and died of multiple organ failure.

## 2. Case presentation

**Figure 1.**
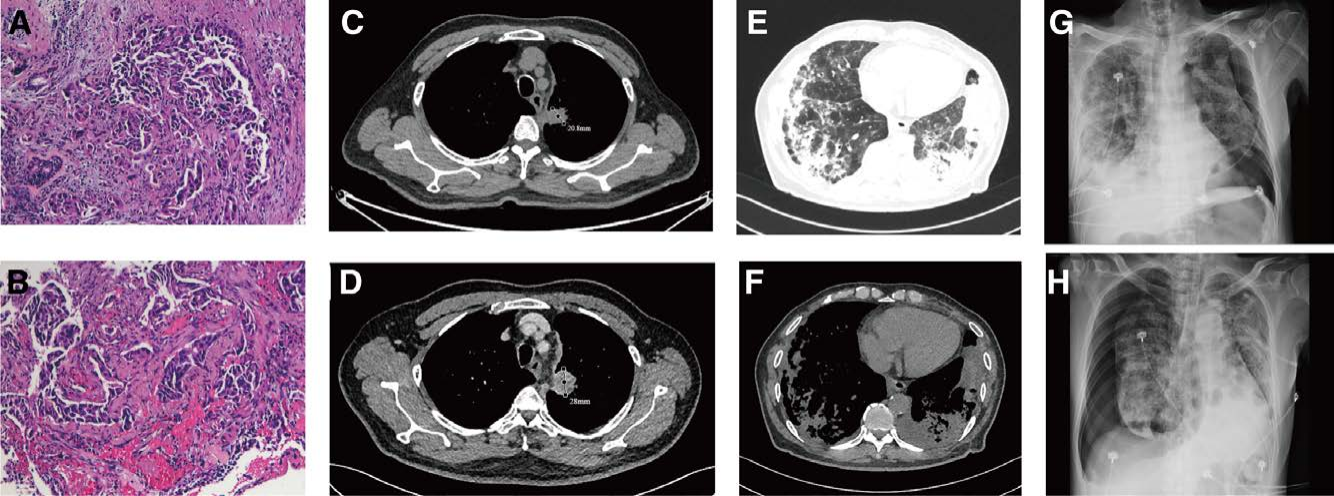
Histology and imaging data. (A and B) Histopathological analysis of the lung tissue specimen. Neoplastic cells are consistent with lung adenocarcinoma. (Hematoxylin and eosin stain at 100× magnification). (C and D) Comparison of the lung adenocarcinoma before and after osimertinib therapy. (C) CT showing a mass with the largest diameter of 28.1nm in lung cancer. (D) The follow-up CT on week 6 of osimertinib therapy revealed that the tumor had shrunk. (E and F) CT scans. CT revealed interstitial lung fibrosis changes in both lungs and pulmonary infiltrates on week 8 of osimertinib therapy. (E) Lung window. (F) Mediastinal window. (G and H) Chest X-ray scans. Chest X-ray revealed severe bilateral pneumothorax. (G) Chest X-ray on August 17, 2020. (H) Chest X-ray on August 19, 2020.

**Figure 2.**
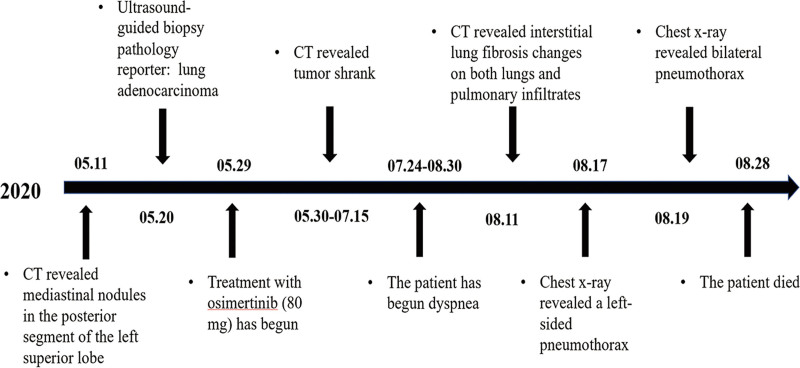
Timeline. The timeline of the medical history of the patient. CT = computed tomography.

A 72-year-old Chinese male patient who presented with progressively worsening cough and sputum for approximately 3 months and poor appetite and fatigue for approximately 2 months was admitted to the hospital on May 8, 2020. The patient was a smoker of 40 years with an 80-pack per year history and had a background of coronary atherosclerotic heart disease and hypertension. Computed tomography (CT) showed mediastinal nodules in the posterior segment of the left superior lobe, measuring 22 × 24*28.1 mm in size, multiple small nodules in the bilateral lung fields, and subcutaneous adipose soft tissue density nodules on the left chest wall (Fig. 1). Head magnetic resonance imaging scan demonstrated the brain metastases. The left chest wall nodules of the patient were not excluded from metastasis. The pathological diagnosis was confirmed by ultrasound-guided surgery of the left chest wall, and the result was lung adenocarcinoma (Fig. 1). Molecular genetic analysis revealed an EGFR mutation L858R in exon 21. Consequently received stage IV (T2NxM1) adenocarcinoma of the left upper lung lobe with a complicated chest wall and brain metastasis diagnosis. Twenty days later, first-line treatment with osimertinib was initiated using a standard dose of 80 mg/day. He initially experienced partial remission of NSCLC for 6 weeks without evidence of gastrointestinal, cutaneous, or cardiac side effects. However, 8 weeks after starting the medication, he started coughing up the phlegm and experienced shortness while exerting himself. Ten weeks after beginning the medicine, the patient was readmitted to the same hospital because the dyspnea and fever symptoms worsened. Computed tomography revealed interstitial lung fibrosis changes in both lungs and pulmonary infiltrates (Fig. 1). After symptomatic treatment failed, the patient was admitted to our hospital for further treatment. Supplemental oxygen was administered because of severe dyspnea. Physical examination revealed the following vital signs: heart rate, 116 bpm; blood pressure, 98/66 mm Hg; O_2_ saturation, 92% while the patient was breathing oxygen, breath rate 33 per minute, and a temperature of 36.3 °C. Bilateral inspiratory crackles were observed in the lower lung field. The arterial blood gas showed a PH of 7.44 (reference range, 7.35–7.45), partial pressure of carbon dioxide of 31.8 mm Hg (reference range, 35–45 mm Hg), and partial pressure of oxygen of 76 mm Hg (reference range, 80–100 mm Hg). Blood work showed remarkably elevated inflammatory parameters (CRP 293.18 mg/dL [standard < 0.5 mg/dL], PCT 0.364 ng/mL, leucocytes 9.34 G/L). D-dimer 1.1 μg/mL. Screening for general bacterial culture, acid-fast Bacillus smear, and culture yielded negative results. No viral or fungal infections were observed. Transbronchial biopsy for histopathological evaluation could not be performed because of the poor performance status. The patient’s medical history was unremarkable, with no history of interstitial lung disease, asthma, COPD, or pulmonary embolism. Furthermore, in addition to osimertinib, no medication was administered in the preceding 10 weeks. We considered that the patient had severe osimertinib-induced pneumonitis. Osimertinib was discontinued, and prednisolone 160 mg/day was prescribed, along with supplemental oxygen and wide-spectrum antibiotics (piperacillin, tazocine, and batavidin combined with moxifloxacin). However, the symptoms did not resolve, and impaired oxygenation persisted over the subsequent 4 days in parallel with persistent pulmonary infiltrates, requiring mechanical ventilation. However, the patient and his family refused mechanical ventilation. On day 5 of hospitalization, rapid aggravation of symptoms of dyspnea was observed; peripheral capillary oxygen saturation was below 80%; the sound of breathing in the left upper lung disappeared; chest radiography was performed immediately, which revealed a left-sided pneumothorax (Fig. 1); and a closed thoracic drainage tube was placed, but his dyspnea did not improve. The patient then underwent endotracheal intubation, assisted ventilation, and continued morphine sedation. The patient’s vital signs were relatively stable. On the seventh day of hospitalization, a right-sided pneumothorax was observed (Fig. 1). A closed thoracic drainage tube was then placed on the right side. Unfortunately, the patient’s general condition deteriorated in the following days, and progressive dyspnea, respiratory failure, and heart failure developed. After 16 days of anti-infection treatment with high-dose methylprednisolone, mechanical ventilation, and supportive care, the patient with severe pneumothorax died of multiple organ failure. At the request of his family, no autopsies were performed. A timeline of the medical history of the patients in our study is presented in Figure 2.

## 3. Discussion

The National Comprehensive Cancer Network NSCLC Panel recommends osimertinib as a preferred first-line therapy option for patients with metastatic NSCLC who have sensitizing EGFR mutations based on a phase 3 trial and FDA approval. Previous reports have described NSCLS patients with EGFR mutations receiving TKI treatment, such as bevacizumab or erlotinib, who presented with symptomatic spontaneous pneumothorax.^[14]^ In fact, spontaneous pneumothorax, a rare adverse event in response to antineoplastic therapy, has been documented in a variety of other tumors, including lung cancer.^[15–18]^ However, the majority of reports involve pneumothorax as a result of treatment for metastatic lung cancer and primary lung cancer following the addition of other treatments.^[19–21]^ After treatment with osimertinib alone, there have been no reports of spontaneous bilateral pneumothorax in patients with primary lung cancer after treatment with osimertinib alone. According to our findings, severe bilateral pneumothorax is an adverse effect of osimertinib treatment. Symptomatic pneumothorax is life-threatening if not recognized and treated effectively.

The mechanism of osimertinib pneumothorax remains unclear; however, some studies have suggested that it is related to tumor necrosis and alveolar injury caused by TKIs. We suggest 4 potential mechanisms underlying TKI pneumothorax. First, TKIs suppress angiogenesis and promote the apoptosis of vascular endothelial cells, and their direct toxicity to type II pneumocytes, tracheal epithelial cells, and vascular endothelial cells leads to pneumothorax.^[22,23]^ A case report showed that biopsy pathology of an EGFR mutation-positive lung adenocarcinoma patient showed extensive necrosis 11.5 months after osimertinib maintenance therapy.^[24]^ The other case report showed that 2 patients with advanced esophageal cancer were treated with oral apatinib; their chest CT showed massive necrosis of tumor tissues, and a cavity was formed locally in each patient.^[25]^ Second, activation of immune system cells may play a key role.^[22]^ A previous clinical study identified that after TKI treatment, inflammatory cytokines such as TNF, IL-1β, GM-CSF, and IL-8 are released, promoting cell apoptosis and enhancing tissue necrosis.^[26–28]^ In addition, a study showed that EGFR-TKIs may enhance the development of pneumonitis in TNF-overexpressing lung tissue through blockade of TNF-induced EGFR transactivation.^[29]^ Third, compression of bronchioles by a tumor causing hyperinflation of the lung segment and rupture of the lung parenchyma and pleural or subpleural lung metastases resulted in pleural traction and pulmonary contracture.^[30]^ Fourthly, some adverse events occur in patients with lung cancer after treatment with TKI, such as ILD and diffuse alveolar hemorrhage, leading to pneumothorax. According to previous studies, severe interstitial inflammation caused by ILD results in fibrocystic hyperplasia and adhesions, fibro-related pleural traction, and pulmonary contractures. In addition, pulmonary fibrosis can dramatically reduce pulmonary compliance and increase the incidence of pulmonary pneumatic injury.^[31]^ The incidence of ILD during TKI treatment has been reported to be 2% to 5%, and patients develop pneumothorax immediately following the onset of ILD.^[32–36]^ In conclusion, we believe that the efficacy and utility of TKI, as well as adverse events resulting from osimertinib, are potential risk factors for the development of pneumothorax.

To date, there are no reports of osimertinib-related pneumothorax. However, a study showed osimertinib combined with anlotinib in EGFRm, treatment-naive advanced NSCLC patients, and pneumothorax accounted for 1/6 of the adverse events.^[37]^ With the widespread application of TKIs, their incidence is expected to increase. One study showed that the presence of cavitary or pleural-based nodules or masses was a significant risk factor for pneumothorax.^[38]^ Additionally, the size of the malignancy (primary or metastatic) and prior history of pneumothorax, emphysema, and cystic lung disease have a higher risk of pneumothorax.^[39]^ Therefore, clinicians should be vigilant regarding TKI-related pneumothorax when TKIs are used in patients with previous lung disease. Along with the disease, poor patient habits may also increase the risk of pneumothorax. Two-thirds of patients with lung tumors had a history of smoking, and lung bullous was a common comorbid disease. Therefore, it is necessary to consider TKI-induced pulmonary bullous ruptures.^[40]^ In addition, based on the biochemical damage caused by TKIs, the primary cause of this might be poor lung tissue tolerance to physical damage. Another study on the safety of repeat lung needle biopsies in patients treated with TKIs showed that pneumothorax occurred in 8.4% of the rebiopsy cases.^[41]^ The type of TKIs chosen was also a potential risk factor for TKI-related pneumothorax. Previous studies have suggested that the incidence of pazopanib-related pneumothorax is approximately 9.1% to 14.6%,^[38,42]^ and that of lenvatinib-related pneumothorax is approximately 2.6%.^[43]^ The type of TKIs chosen was also a potential risk factor for TKI-related pneumothorax. Reviews of risk factors for TKI-related pneumothorax will facilitate the early diagnosis and management of high-risk groups.

Furthermore, we are still unsure when TKI-related pneumothorax first appears. A 2016 single-institute analysis suggested that pneumothorax occurred as early as the 6th day after pazopanib treatment in patients with soft tissue sarcoma.^[44]^ Another study showed that in the seventh month of pazopanib therapy, a patient with high-grade endometrial stromal sarcoma developed pneumothorax.^[45]^ But a case report showed that lung adenocarcinoma patients develop pneumothorax after 4 months of anlotinib treatment.^[40]^ Therefore, it is challenging to make predictions about when a TKI-related pneumothorax will manifest based on the data that is currently available in the literature. Further clinical statistics are required to assist clinicians in this regard.

After 8 weeks of osimertinib treatment, the patient experienced coughing, expectoration, and dyspnea. After 10 weeks of osimertinib treatment, restaging CT revealed ILD and bilateral pneumothorax. Thus, we hypothesized that drug toxicity caused the pneumothorax. For patients with pneumothorax, needle aspiration, and intercostal drainage are first-line therapies.^[46]^ For the treatment of drug-induced adverse events, This problem resolves with the discontinuation of medication and systemic glucocorticoids for the treatment of drug-induced adverse events.^[47]^ Our patient received osimertinib therapy, and pneumothorax did not occur until after 10 weeks of treatment. Owing to severe pneumothorax, osimertinib rechallenge was not appropriate. After discontinuing osimertinib and receiving glucocorticoid and thoracic close drainage therapy, his symptoms did not improve. In addition, it was difficult to correct the severe bilateral pneumothorax; the patient’s family refused mechanical ventilation, and the patient ultimately died. We believe that patients receiving osimertinib or other EGFR-TKIs who have severe dyspnea should use invasive ventilator-assisted ventilation as soon as possible, with the permission of the patient and family members.

## 4. Conclusions

Notably, to our knowledge, this is the first report of severe bilateral pneumothorax during osimertinib treatment. Based on previous reports and our own experience, we suggest that osimertinib-treated patients with lung cancer have metastatic tumors of the pleura, prior history of emphysema, cystic lung disease, ILD, old age, and smoking. At this time, imaging changes in the lung should be monitored instead of waiting for obvious aggravation of symptoms. The drug should be stopped promptly, and appropriate treatment should be used to reduce the fatality rate. In addition, this case also suggests that clinicians should be aware of the possibility of pneumothorax and pay attention to the changes in chest and vital signs at any time to prevent misdiagnosis and missed diagnosis, and delay the disease when patients with lung cancer suffer from acute respiratory distress during osimertinib administration.

## Acknowledgments

We wish to thank those whose work could not be cited due to space limitations for their timely help.

## Author contributions

**Investigation:** He Li, Xiaojuan Shi, Dongchang Wang.

**Methodology:** He Li, Xiaojuan Shi, Dongchang Wang.

**Writing – original draft:** He Li.

**Writing – review & editing:** Gang Chen, Dongchang Wang.
